# Polymer Nanocomposites with High Energy Density Utilizing Oriented Nanosheets and High-Dielectric-Constant Nanoparticles

**DOI:** 10.3390/ma14174780

**Published:** 2021-08-24

**Authors:** Yushu Li, Yao Zhou, Sang Cheng, Jun Hu, Jinliang He, Qi Li

**Affiliations:** State Key Lab of Power Systems, Department of Electrical Engineering, Tsinghua University, Beijing 100084, China; liyushu16@mails.tsinghua.edu.cn (Y.L.); zhouyao6811@163.com (Y.Z.); chengs1995@126.com (S.C.); hjun@tsinghua.edu.cn (J.H.); hejl@tsinghua.edu.cn (J.H.)

**Keywords:** discharged energy density, charge–discharge efficiency, electric breakdown, ferroelectric films, dielectric losses

## Abstract

The development of high-energy-density electrostatic capacitors is critical to addressing the growing electricity need. Currently, the widely studied dielectric materials are polymer nanocomposites incorporated with high-dielectric-constant nanoparticles. However, the introduction of high-dielectric-constant nanoparticles can cause local electric field distortion and high leakage current, which limits the improvement in energy density. In this work, on the basis of conventional polymer nanocomposites containing high-dielectric-constant nanoparticles, oriented boron nitride nanosheets (BNNSs) are introduced as an extra filler phase. By changing the volume ratios of barium titanate (BT) and BNNSs, the dielectric property of polymer nanocomposites is adjusted, and thus the capacitive energy storage performance is optimized. Experimental results prove that the oriented BNNSs can suppress the propagation of charge carriers and decrease the conduction loss. Using poly(vinylidene fluoride-co-hexafluoropropylene) (P(VDF-HFP)) as the polymer matrix, the P(VDF-HFP)/BNNS/BT nanocomposite has a higher discharged energy density compared with the conventional nanocomposite with the freely dispersed BT nanoparticles.

## 1. Introduction

Dielectric capacitors are widely used as energy storage and conversion devices in electrical systems and advanced electronics, such as power inverters, medical defibrillators, pulse forming networks, hybrid electric vehicles, and portable electronics [1,2,3,4]. Among the various categories of dielectric capacitors, polymer film capacitors based on organic dielectric polymer films are preferred due to the advantages of graceful failure mechanism, light weight, processing flexibility, low cost, high breakdown strength, and simple integrated assembly process [5,6]. However, the relatively low energy density remains a major challenge and impedes the further application of polymer capacitors. For instance, the energy density of the state-of-the-art polymer dielectrics, biaxially oriented polypropylenes (BOPPs), is only 1~2 J/cm^3^, an order of magnitude lower than that of electrochemical capacitors (20–30 J/cm^3^) [7].

In practical applications, the repetition rate of charge–discharge cycles must be increased in order to compensate for the low energy density of polymer film capacitors, which would lead to accelerated heating, fast aging, and decreased reliability. Due to the low thermal conductivity of polymers, the internal temperature of the capacitors would rise rapidly, resulting in thermal runaway eventually [8,9]. Therefore, it is of great significance to improve the energy density of polymer dielectrics to secure the stability and to reduce the volume and cost of the capacitors.

Under the external electric field, the stored energy density in dielectrics can be expressed as *U* = ∫*E*d*D*, where *U* is the energy density, *E* is the applied electric field, and *D* is the electrical displacement. In a linear dielectric material, *D* increases monotonically with increased external electric field *E* and dielectric constant *ε* of the dielectric, indicating that elevation of both *ε* and breakdown strength (*E*_b_) can lead to higher energy density. Polymer dielectrics generally have high *E*_b_, but the relatively low *ε* limits their energy density. For example, although the breakdown strength of BOPP films is as high as ~700 MV/m, the low dielectric constant of ~2.2 restricts the energy density to only ~5 J/cm^3^ [10].

Among the polymer dielectrics, ferroelectric polymers are promising dielectric materials for energy storage applications, benefiting from the combination of relatively high *ε* and high *E*_b_ [11]. To further improve the energy density of ferroelectric polymers, extensive studies have been carried out to further increase *ε* by adding high-dielectric-constant nanofillers [12], such as barium titanate (BaTiO_3_), barium strontium titanate (Ba_x_Sr_1−x_TiO_3_), and lead zirconate titanate (Pb(Zr,Ti)O_3_) [13,14,15]. However, since nanofillers usually have high dielectric constant and high conductivity, the introduction of nanofillers will cause local electric field distortion of the nanocomposites and increase leakage current and loss [16]. As a result, the introduction of high-dielectric-constant nanofillers cannot significantly increase the discharged energy density. Alternatively, various highly insulated nanofillers, such as boron nitride (BN) [17,18], silicon oxide (SiO_2_) [10], and alumina (Al_2_O_3_) [19], have been used to improve *E*_b_. These nanofillers serve as barriers in nanocomposites to prolong the transport path of charge carriers and effectively inhibit the growth of electrical trees, thus increasing the breakdown strength [20,21,22].

In this study, we demonstrate a ternary nanocomposite composed of high-dielectric-constant BaTiO_3_ (BT) nanoparticle and oriented two-dimensional boron nitride nanosheet (2D-BNNS), which alleviates the drawbacks of free dispersed BT nanoparticles, retains the excellent insulating property of BNNSs, and realizes the simultaneously enhanced ε and *E*_b_. Furthermore, the BNNS is oriented by a doctor blade to better impede the charge carrier transport [23], which is advantageous in comparison to the randomly dispersed fashion [24], implying that the same effectiveness in suppressing energy loss can be achieved at a lower filler content. The dielectric constant, breakdown strength, and discharged energy density of the resultant nanocomposites can be adjustable by simply tuning the content and ratio of BT and BNNS. It was found that the nanocomposites with the optimal content of nanofillers discharged an energy density as high as 13.0 J/cm^3^ with the charge–discharge efficiency of 72% under an electrical field of 547 MV/m.

## 2. Materials and Methods

### 2.1. Materials

P(VDF-HFP) copolymer was obtained from Poly*K* Technologies. BT nanoparticles with an average diameter of about 50 nm and hexagonal boron nitride (*h*-BN) powder with an average diameter of about 10 μm were purchased from Sigma-Aldrich. N,N-Dimethylformamide (DMF) was supplied by Aladdin Co., Shanghai, China.

### 2.2. Exfoliation of BNNSs

BNNSs were obtained by an ultrasound-assisted peeling method [17]. The exfoliation process of *h*-BN powder was similar to our previous work. First, 2 g of *h*-BN powder was dispersed in a 300 mL of DMF solution. Then, the mixed solution was tip-sonicated (700 W, amplitude 70%) for 24 h. The obtained solution was centrifuged at 1000 rpm for 40 min. The supernatant was subsequently centrifuged at 10,000 rpm for another 40 min, and the sediment was collected. Finally, the sediment was dried at 70 °C for 24 h in a vacuum oven, and then BNNSs were obtained.

### 2.3. Fabrication of P(VDF-HFP)/BNNS/BT Nanocomposites

Five hundred milligrams of P(VDF-HFP) (90/10) powder was dissolved in 10 mL DMF and stirred for 12 h. A solution of 50 mg/mL was prepared. BNNSs were evenly dispersed in DMF to obtain a solution of 4 mg/mL. The above solution was sonicated for about 1 h by tip-type sonication (700 W, amplitude 60%). The resultant solution was poured into P(VDF-HFP) solution, followed by stirring vigorously for 12 h. BT nanoparticles were then added, and the mixture was sonicated (700 W, amplitude 60%) for 30 min. The sonicated mixture was cast onto the glass sheet by an ink scraper to align the BNNSs in a horizontal direction. It was dried in a blast oven at 40 °C for 6 h and then in a vacuum oven at 40 °C for 12 h. Finally, after heating at 200 °C for 5 min, the film was immediately quenched in cold water in order to increase the nonpolar γ-phase in the polymer matrix and then peeled off from the glass plate. Finally, the films were dried at 40 °C in vacuum overnight. The typical thickness of the P(VDF-HFP)/BNNS/BT nanocomposites was around 15 μm.

### 2.4. Characterization

Fourier transform infrared (FT-IR) spectroscopy was performed on a Thermo Scientific Nicolet iS10 spectrometer at 4 cm^−1^ resolution in the attenuated total reflectance (ATR) mode. X-ray diffraction (XRD) analysis was conducted on a D/max-2500/PC with Cu Kα radiation (λ = 0.154 nm). The scanning range was 5–80°, and scanning rate was 2°/min. Scanning electron microscopy (SEM) images of all samples were obtained with a Hitachi SU8010 field emission microscope. Atomic force microscopy (AFM) images of all samples were obtained with a Bruker Dimension Icon atomic force microscope in tapping mode. Leakage current density was measured under an electric field (100 MV/m) provided by a Hewlett Packard 4140B Picoammeter/voltage source. Dielectric constant and dissipation factor were measured on a Novocontrol Concept 80 dielectric spectroscopy meter at room temperature. Dielectric breakdown strength measurements were collected with a TREK 610C amplifier. The DC voltage ramp was 500 V/s, and the limit current was 5 mA. In this study, the experiment data of dielectric breakdown strength measurements were analyzed by two-parameter Weibull statistics, *P*(*E*) = 1 − exp(−(*E*/*α*)*β*), where *α* is the scale parameter denoting the electric field strength, for which there is a 63% probability of breakdown (Weibull breakdown strength), and *β* is the shape parameter representing the scatter of data. High-field *P*-*E* loops were collected by a ferroelectric test system (Poly*K* Technologies), where the samples were subjected to a triangular unipolar wave with a frequency of 10 Hz. Gold electrodes of about 60 nm thickness were sputtered on both sides of the polymer films for electrical measurements. For the breakdown strength, leakage current, *D*-*E* loop, and dielectric spectrum, the diameters of gold electrodes were 2.6, 3, 3, and 20 mm, respectively.

## 3. Results and Discussion

### 3.1. Structure and Morphology Characterization

**Figure 1:**
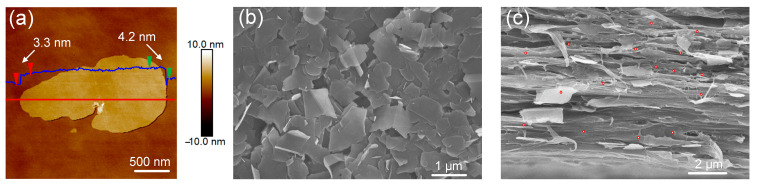
(**a**) AFM image of BNNSs and section analysis along the red line showing the sheet thickness. SEM images of (**b**) BNNSs and (**c**) cross-sectional SEM image of the ternary nanocomposite films containing 6 vol.% BNNS and 3 vol.% BT. The location of some BT nanoparticles in SEM images is circled in red.

The BNNSs were obtained by an ultrasound-assisted peeling method, and the typical AFM image of the individual freestanding BNNS shows a distinct 2D lamella structure. From Figure 1a,b, the typical BNNS has a lateral size of 1–2 μm and a thickness of 3–4 nm. The thinner thickness of the BNNS guarantees higher breakdown strength, and the larger lateral size of the BNNS extends the charge carrier channel and reduces the charge mobility. The BT and BNNSs were randomly dispersed on the P(VDF-HFP) matrix by solution blending. To make BNNSs perform better in improving the insulating properties, the shear force of the doctor blade was used to align the BNNSs in the nanocomposite in the film direction. Figure 1c shows the cross-sectional SEM images of the ternary nanocomposite films. No heavy agglomeration of BT nanofillers was seen, and most BNNSs were oriented in the film direction.

**Figure 2:**
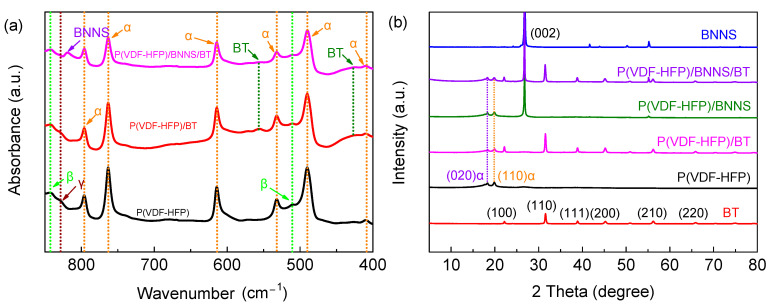
(**a**) FT-IR spectra of pure P(VDF-HFP), P(VDF-HFP)/BT, and P(VDF-HFP)/BNNS/BT. (**b**) XRD patterns of the BNNS, BT, pure P(VDF-HFP), P(VDF-HFP)/BNNS, P(VDF-HFP)/BT, and P(VDF-HFP)/BNNS/BT.

Figure 2a illustrates the FT-IR spectra of pure P(VDF-HFP), P(VDF-HFP)/BT binary nanocomposites, and P(VDF-HFP)/BNNS/BT ternary nanocomposites. The absorption bands at 428 and 550 cm^−1^ correspond to metal–oxygen vibrations and the stretching vibrations in the TiO_6_ octahedra, respectively, of BT [25]. The signal peak at 818 cm^−1^ belongs to BNNS. The absorption bands at 796, 763, 614, 532, 490, and 410 cm^−1^ belong to the *α*-phase in P(VDF-HFP), while those at 840 and 511 cm^−1^ are ascribed to the *β*-phase. The absorption band at 834 cm^−1^ belongs to the *γ*-phase in P(VDF-HFP) [26]. It is found that the incorporation of nanofillers may not influence the crystal phase of P(VDF-HFP), and no detectable chemical interaction between the nanoparticles and polymer matrix is observed. Figure 2b shows that the XRD patterns of the BNNS, BT, pure P(VDF-HFP), P(VDF-HFP)/BNNS, P(VDF-HFP)/BT, and P(VDF-HFP)/BNNS/BT, where the peaks at 18.3 and 19.9° are ascribed to (020)_α_ and (110)_α_ faces, respectively [27,28]. The result indicates that the P(VDF-HFP) polymer is mainly of a nonpolar phase, which is favorable for dipolar switching and thus reduced hysteresis loss. Table 1 summarizes that the mean size of crystallite for (020)_α_ and (110)_α_ of pure P(VDF-HFP) and the nanocomposites. Results indicate that the incorporation of BNNS and BT decreases the crystallite size of the polymer and thereby reduces the hysteresis loss.

### 3.2. Electrical Performance of the Nanocomposites

**Figure 3:**
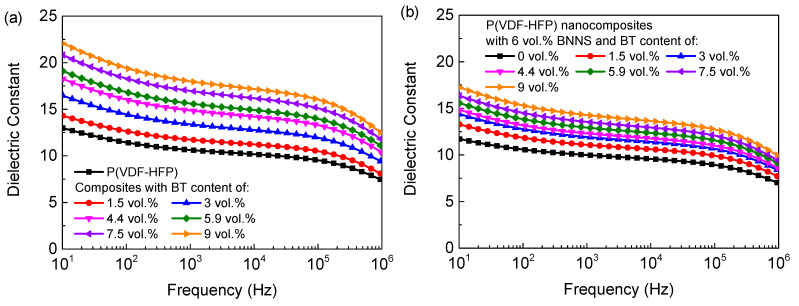
Frequency-dependent dielectric constant of (**a**) the P(VDF-HFP)/BT nanocomposites and (**b**) the ternary nanocomposites with 6 vol.% BNNSs.

**Figure 4:**
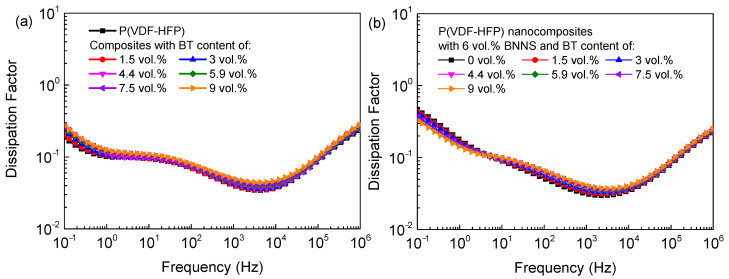
Frequency-dependent dissipation factor of (**a**) the P(VDF-HFP)/BT nanocomposites and (**b**) the ternary nanocomposites with 6 vol.% BNNSs.

In order to simultaneously improve the electrical breakdown strength and dielectric constant of P(VDF-HFP), a library of ternary nanocomposites with fixed content of BNNSs and varied content of BT were prepared. The frequency-dependent dielectric constant is shown in Figure 3. As the BT content gradually increased to 9 vol.%, the dielectric constant of the binary nanocomposites increased from 10.6 to 17.9 (Figure 3a) at 1 kHz due to the higher dielectric constant of BT relative to pure P(VDF-HFP) (~3000 vs. ~12). The frequency-dependent dissipation factor of the binary and ternary nanocomposites are shown in Figure 4. The dissipation factor of the binary nanocomposites increased slightly with respect to the pure polymer, e.g., the dissipation factor of the binary nanocomposites with 9 vol.% BT is 0.049 at 1 kHz, lower than that of pure P(VDF-HFP), 0.039. To achieve simultaneously high dielectric constant and low dielectric loss, the oriented BNNSs with superior insulation performance were incorporated into the nanocomposites with randomly dispersed BT particles. With the introduction of the oriented BNNSs, the dissipation factor of the ternary nanocomposites substantially decreased, and dielectric constant of the P remained relatively high. For example, the dissipation factor of the ternary nanocomposites with 6 vol.% BNNSs and 9.8 vol.% BT was only 0.040 at 1 kHz, 18% lower than that of the binary nanocomposites. Moreover, the ternary nanocomposites still exhibited a higher dielectric constant than pure P(VDF-HFP). At high frequency, the dielectric relaxation is called *α*_a_-relaxation, which is related to segmental molecular motions in the amorphous of the polymer matrix. At intermediate frequency, the relaxation peak is called *α*_c_-relaxation, which is associated with the molecular motion in the crystalline phase of the polymer matrix. At low frequency, the dielectric relaxation is related to the charge migration of the polymer matrix. Interestingly, after doping with horizontally oriented BNNSs, the relaxation peak of charge migration of nanocomposites was significantly suppressed, even lower than that of pure P(VDF-HFP), showing that the horizontally oriented BNNSs may block charge migration and reduce ion conduction loss.

**Figure 5:**
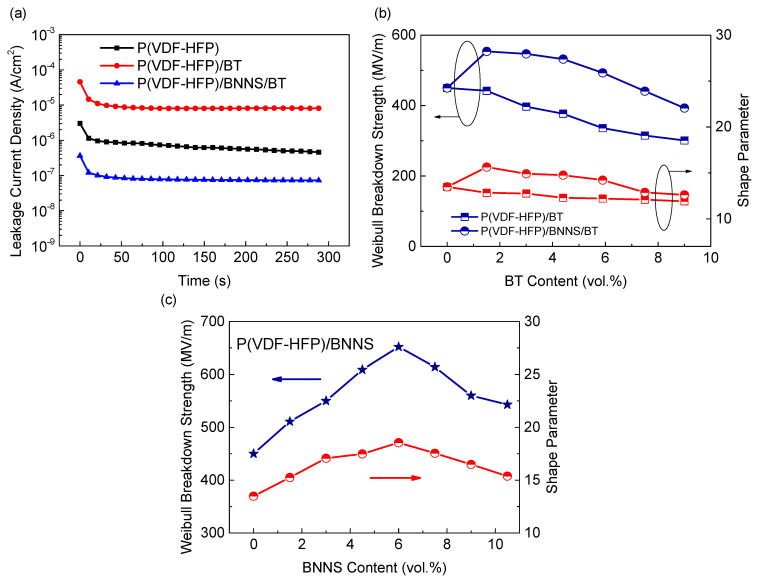
(**a**) Leakage current density of P(VDF-HFP), P(VDF-HFP)/BT, and P(VDF-HFP)/BNNS/BT nanocomposites under the electric field of 100 MV/m. The characteristic breakdown strength and shape parameter of (**b**) P(VDF-HFP)/BT and P(VDF-HFP)/BNNS/BT nanocomposites as a function of BT content and (**c**) P(VDF-HFP)/BNNS nanocomposites as a function of BNNS content.

The dissipation factor only represents the dielectric loss under low electric fields, but the capacitor films usually operate under high electric fields. Figure 5a shows the leakage currents of pure P(VDF-HFP), binary nanocomposites, and ternary nanocomposites under an electric field of 100 MV/m. It is clear that the introduction of BT resulted in a significant increase of leakage current in the binary nanocomposites; i.e., the leakage current density increased from 4.6 × 10^−7^ A/cm^2^ for pure P(VDF-HFP) to 8.1 × 10^−6^ A/cm^2^ for the binary nanocomposites, which is nearly one order of magnitude higher. Conversely, the introduction of oriented BNNSs could effectively suppress the leakage current. The leakage current of the ternary nanocomposites (i.e., 7.3 × 10^−8^ A/cm^2^) is two orders of magnitude lower than that of the binary nanocomposites. It is also even much lower than that of the pure P(VDF-HFP). Therefore, the oriented BNNSs not only suppress the dielectric loss under low electric fields but also reduce the leakage current under high electric fields in P(VDF-HFP) nanocomposites.

Apart from high dielectric constant and low conduction loss, high electrical breakdown strength of polymer dielectrics is highly desired to pursue high energy density. The electrical breakdown strength of the binary nanocomposites and the ternary nanocomposites as a function of BT content are summarized in Figure 5b. It is evident that the introduction of BT led to significantly reduced breakdown strength from 450 MV/m for pure P(VDF-HFP) to 301 MV/m for the binary nanocomposites with 9 vol.% BT, indicating a reduction of about 33%. The decreased breakdown strength in the binary nanocomposites is associated with the higher electrical conductivity of BT and large distinction in dielectric constant between BT and P(VDF-HFP) matrix, making BT act as defects in the nanocomposites. Moreover, the poor compatibility between BT nanoparticles and P(VDF-HFP) matrix leads to loose-bounded interfaces. So, more voids and flaws are introduced at the interfaces, and thus the breakdown strength is reduced. To improve the breakdown strength, BNNSs were oriented perpendicular to the electric field direction to impede the charge transport and breakdown pathway. It can be seen that the incorporation of oriented BNNSs dramatically promoted the breakdown strength of nanocomposites (Figure 5c). For example, the breakdown strength of the ternary nanocomposites with 6 vol.% BNNS and 5.9 vol.% reached 493 MV/m, 47% higher than that of the binary nanocomposites, ~336 MV/m. Additionally, the ternary nanocomposites exhibited higher *β* values than the binary nanocomposites, indicating less scatter of breakdown strength for the ternary nanocomposites. The variation in breakdown strength coincides well with the variation in the leakage current, showing that the suppressed leakage current is responsible for the increased breakdown strength.

### 3.3. Energy Storage Performance of the Nanocomposites

**Figure 6:**
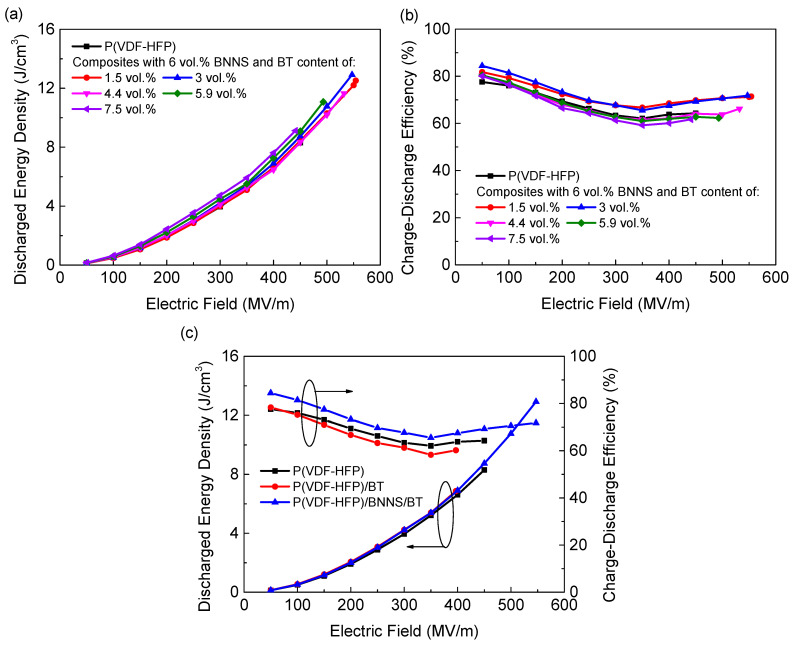
(**a**) Discharged energy density of the ternary nanocomposites with 6 vol.% BNNSs and various content of BT. (**b**) Charge–discharge efficiency of the ternary nanocomposites with 6 vol.% BNNSs and various content of BT. (**c**) Discharged energy density and charge–discharge efficiency of pure P(VDF-HFP), binary nanocomposites, and ternary nanocomposites under different electric fields.

The energy storage properties of the nanocomposites are characterized by charge–discharge efficiency (*η*) and discharged energy density (*U*_e_). It was seen that the ternary nanocomposites showed significantly promoted maximum *U*_e_ compared to pure P(VDF-HFP) due to simultaneously enhanced *ε* and *E*_b_ (Figure 6a). *η* of the ternary nanocomposites remained relatively high at various loads of BT nanoparticles since the insulating networks formed by oriented BNNSs would act as charge barriers and lead to lower conduction loss (Figure 6b). In comparison, although the polarization of the binary nanocomposites was enhanced, little changes could be found in *U*_e_ compared to pure P(VDF-HFP) due to the higher energy loss of the binary nanocomposites (Figure 6c). The optimal composition with a maximum *U*_e_ was determined as the ternary nanocomposite incorporated with 6 vol.% BNNS and 3 vol.% BT. Owing to the high efficiency induced by the incorporation of oriented BNNSs, the ternary nanocomposites delivered a maximum *U*_e_ of 13 J/cm^3^, 57% and 89% higher than pure P(VDF-HFP) and the binary nanocomposites with the same content of BT, respectively.

## 4. Conclusions

In summary, this work shows that the incorporation of oriented BNNSs in P(VDF-HFP) nanocomposites leads to an enhancement in both charge–discharge efficiency and discharged energy density. By changing the volume ratio of BT and BNNS, the dielectric properties of nanocomposites can be continuously adjusted and optimized. The best energy storage properties of the P(VDF-HFP)/BNNS/BT ternary nanocomposites were rationalized experimentally. The concept proposed in this paper can be applied to other ferroelectric nanocomposites to optimize capacitive performance.

## Figures and Tables

**Table 1:** The mean size of crystallite for (020) and (110) faces of pure P(VDF-HFP) and the nanocomposites, from the XRD results.

Filler	D_(020)α_	D_(110)α_
0 vol.% BNNS + 0 vol.% BT	17.37	17.61
6 vol.% BNNS + 0 vol.% BT	16.26	16.88
6 vol.% BNNS + 1.5 vol.% BT	15.48	16.86
6 vol.% BNNS + 3 vol.% BT	15.44	16.57
6 vol.% BNNS + 4.4 vol.% BT	15.23	16.48
6 vol.% BNNS + 5.9 vol.% BT	14.87	16.40
6 vol.% BNNS + 7.5 vol.% BT	14.73	15.38
6 vol.% BNNS + 9 vol.% BT	14.53	15.12

## Data Availability

The data presented in this study are available on request from the corresponding author.
